# Sprout Regeneration of Shrub Willows after Cutting

**DOI:** 10.3390/plants9121684

**Published:** 2020-12-01

**Authors:** Yang Zou, Xiaoping Li, Guo Yang

**Affiliations:** 1 The Key Laboratory of Tree Genetics and Biotechnology of Jiangsu Province and the Education Department of China, College of Forest, Nanjing Forestry University, Nanjing 210000, China; zouyang@njfu.edu.cn (Y.Z.); gbz@njfu.edu.cn (X.L.); 2 School of Life Science, Shaoxing University, Shaoxing 312000, China

**Keywords:** stump size, sprout regeneration, biomass, short-rotation coppice, willow

## Abstract

Shrub willow (*Salix* L. spp.) is a promising bioenergy resource crop due to its high growth rates and superb regenerative ability. Sprouting capacity is influenced by many factors, such as parent tree species and size, which are important limiting factors for stump survival or sprout growth. In this study, we aimed to quantify the survival and regeneration performance of sprouts (including sprout height, sprout diameter, sprout number, leaf morphological traits, leaf chlorophyll content, and ground part dry biomass) from the stumps of two *Salix* species from three diameter classes (10–15, 16–19, and 20–30 mm). An attempt was made to explore why the stump size affects the regeneration of willows by analyzing the carbon and nitrogen proportion of stumps. Stump survival did not differ between the two *Salix* species. However, the sprout regeneration of *S. triandra* was much better than that of *S. suchowensis.* An increase in stump diameter caused increases in the number of sprouts produced per stump, the mean height and basal diameter of sprouts per stump, the leaf chlorophyll content, and the biomass of sprouts per stump. By contrast, stump diameter did not significantly affect stump survival. The results indicate that the larger stumps store more carbon and nitrogen than small-sized stumps, which may be one of the reasons why the larger willow stumps have a stronger resprouting ability. This study provides essential information regarding the sprout regeneration of short-rotation coppice willow plantations after harvest.

## 1. Introduction

The current main sources of liquid biofuels are traditional crops. These so-called first-generation biofuels have come under intense criticism for competing with food production [1]. Lignocellulosic materials in the form of plant stems and leaves represent a rich source of reduced carbon that could be used to produce second-generation biofuels. The term short-rotation coppice (SRC) refers to biomass production systems cultivated for energy purposes by using fast-growing tree species with the ability to regenerate from the stumps after harvest. SRC requires less operational effort and nutrients and has a more positive effect on soil ecology and biodiversity compared to annual crops [2,3]. With the expansion of the demand for bioenergy and the demand for wood biomass, SRC is becoming increasingly important around the world [4]. Willow (*Salix* L. spp.) and poplar (*Populus* L. spp.) are the most common species currently used in commercial SRC systems. Shrub willows have several traits, including high yields, easy vegetative propagation, a short reproduction cycle, and the ability to regenerate after multiple harvests, making them ideal for SRC systems [5,6,7,8]. Thus, shrub willow has been widely studied as a dedicated bioenergy crop in SRC systems.

Willows have a strong sprouting ability. Sprouts produced from the stump are the most important source of regeneration for willows after cutting. They have a large established root system, which usually supports rapid growth, multiple flushes from stored nutrients, and the increased ability to obtain resources [9,10]. The potential of tree stumps to survive and sprout after cutting is influenced by many factors, such as the age of the parent tree [11,12,13], the harvesting season [14], and the density of remaining trees and nearby stumps [11,15]. Stump diameter is also a crucial factor. In one study, stump diameter and height positively influenced the sprout number and sprout height of *Quercus variabilis* during the first year, and the effect vanished in the second and third years [14]. Effects of parent size and neighborhood density on resprouting and the effects on stump survival and sprout growth after logging were recently explored in detail in a paper by Matula et al. [16]. Several studies of birch have revealed positive relationships between stump diameter and stump survival and sprout growth [17,18]. However, few studies on the regeneration ability of willows after harvest have been previously reported. Consequently, information on the effects of willow stump diameter on sprout survival and growth is lacking.

Carbon and nitrogen storage play an important role in regulating tree growth [19,20]. Previous studies have shown that the growth of trees can be dependent on external and internal sources of carbon and nitrogen. External nitrogen sources include the absorption of soil nitrogen by roots, and an external carbon source is the assimilation of carbon dioxide from the atmosphere through photosynthesis [21]. The internal sources of plants come from the storage of carbon and nitrogen [22]. Total non-structural carbohydrates and total non-structural nitrogen reserves could affect plant growth [23,24,25]. How the diameter of stumps is able to influence the survival and regeneration performance of sprouts is a valuable scientific question, but currently studies lack data in this area. It is not known if the diameter of stumps is related to stored carbohydrate and nitrogen. Thus, the mechanism of the phenomena reported here is still not clear.

*Salix suchowensis* and *Salix triandra* are shrub willows with relatively different ecological habits, distributed in Central and Northern China, respectively. These two kinds of shrub willow are considered promising biomass crops due to their superb coppicing capacity [26]. In this study, we aimed to investigate the effect of stump diameter on the regeneration of shrub willows and to analyze how the diameter of stumps could influence sprout survival and regeneration performance.

## 2. Materials and Methods

### 2.1. Plant Materials and Field Trial Design

Two full-sib families were established for *S. suchowensis* by crossing NF2 and XY12 with LS7 separately. A full-sib pedigree was established for *S. triandra* through a DB447 × DB134 cross. The cutting orchard for these pedigrees was maintained at Sihong Forest Farm in Jiangsu Province, China. In the spring of 2018, over 7000 cuttings were collected from 787 progenies of three willow families planted using a random block design. The planting density was 0.5 m × 0.5 m. At the end of 2018, the willow shoots at 5 cm above the ground in the two blocks were harvested with a branch shear. The stumps were classified using diameter into three groups, namely, 10–15, 16–19, and 20–30 mm. 

The field test was implemented at Baima Forest Farm in Nanjing, Jiangsu Province, China (N 31° 60′ E 119° 17′). The average annual temperature is 15.4 °C and the average annual precipitation is 1009.7 mm. The soil on this site was yellow-brown loam (pH, 6.0; organic matter, 20.4 g kg^−1^; total nitrogen, 1.3 g kg^−1^; available phosphorus, 11.5 mg kg^−1^; available potassium, 135.3 mg kg^−1^). 

### 2.2. Measurement of Growth Traits

In November 2019, the sprout number per stump was measured. The height of all the willow sprouts per stump was also measured using a tape ruler with an accuracy of 1 mm. The basal diameters of all the shoots per stump were measured at 5 cm above the ground by using a Vernier caliper with a precision of 0.1 mm. A leaf area meter (YMJ-B, Top, Zhejiang) was used to measure the leaf areas of the fresh leaves from the bottom, middle, and top of canopies at the end of September 2019. The SPAD (Soil and Plant Analyzer Development) value representing absorbance by chlorophyll was measured with a chlorophyll meter TYS-B (Top, Zhejiang). Three fresh leaves of each plant were selected for measurement at the end of September 2019. Moreover, 60 progenies were randomly picked from each willow family. The willow shoots at 5 cm above the ground were harvested with a branch shear at the end of November 2019. The harvested shoots were dried using a DHG-9140A electric heating air blast drying box (Bo xun, Shanghai) at 105 °C until constant weight. The dry weight of each shoot was measured using an electronic balance with a precision of 0.1 g.

### 2.3. Carbon and Nitrogen Content of Stumps

At the end of November 2018, 60 progenies were randomly picked and harvested from each willow family. A 5-cm sample was collected from the lower part of the stem. The stem samples with bark were dried using a DHG-9140A electric heating air blast drying box, then ground to a wood meal. We measured the nitrogen and carbon concentration of the stump samples using an Element analyzer (Vario EL cube, Elementar Corporation, Langenselbold, Germany) in 5 mg of the wood meal of the stumps, according to the manufacturer’s directions [27].

### 2.4. Data Analysis

Growth trait data were analyzed using the linear mixed model on SPSS 23.0 statistical software (IBM Corp, Armonk, NY, USA). The model used was as follows:

*y_ijk_ = µ + α_i_ + β_j_ + λ_k_ + (αβ) _ij_ + e_ijk_,*

where *y_ijk_* is the observed value of the response variable (i.e., sprout height, sprout diameter, sprout number, leaf width, leaf length, leaf area, leaf chlorophyll content, and dry biomass) of species *i* in block *k*, considering stump diameter level *j*; *µ* is the overall mean; *α_i_*, *β_j_*, and *λ_k_* are the species, stump diameter, and block effects, respectively; *(αβ)_ij_* is the bifactorial interactions; and *e_ijk_* is the random experimental error term. Species and stump diameter were considered as fixed effects, whereas block and error were considered as random effects. The linear mixed model was used to evaluate the significance of the effects of the factors and any interaction. Tukey’s honestly significant difference multiple comparison test was used to identify significant differences among basal diameter classes (*p* < 0.05).

The results were represented as mean ± SE (standard error). The correlations of stump diameter with the growth traits of willows were tested using Pearson’s correlations. Boxplots were made using the ggplot2 package in R software (version 3.6.0).

## 3. Results

### 3.1. Effect of Stump Diameter on Shoot Survival

A total of 3900 and 780 stumps were retained in *S. suchowensis* and *S. triandra*, respectively. Among them, 1380, 1743, and 1557 stumps were included in three diameter classes of 10–15, 16–19, and 20–30 mm, respectively (Table 1). The lowest survival was 94.3%, which was observed in *S. triandra* stands. The highest survival was 98.8% (Table 1), which was observed in *S. suchowensis* stands. The stump diameter class had no significant effect on stump survival.

### 3.2. Effect of Stump Diameter on Shoot Growth Traits

**Figure 1:**
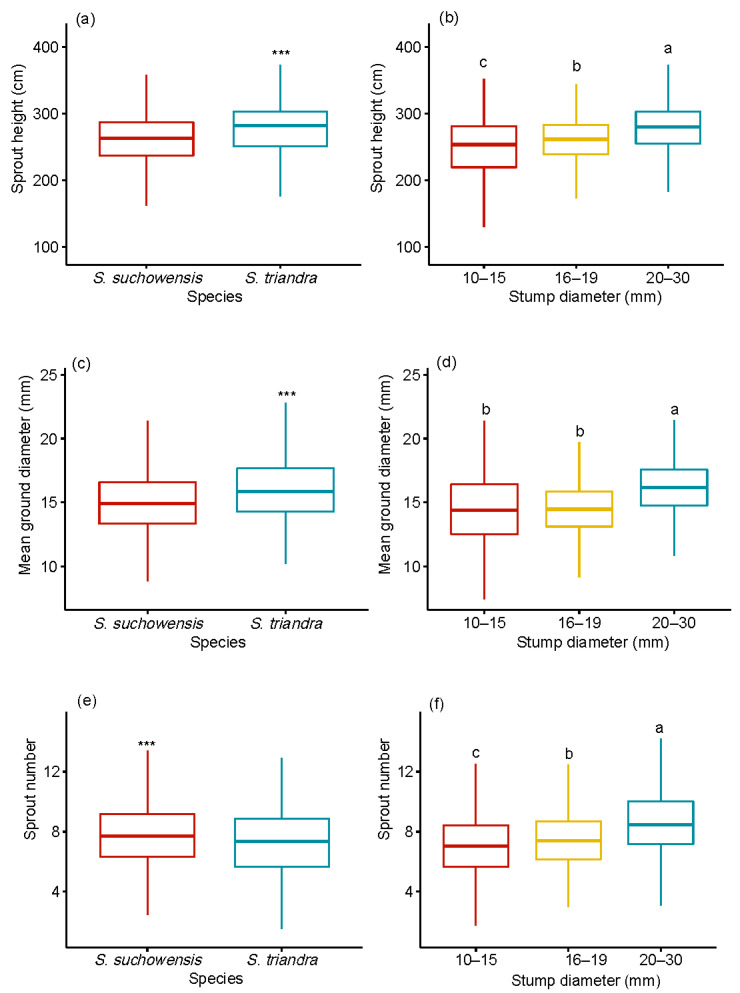
Effect of stump diameter and species on sprout height (**a**,**b**), sprout basal diameter (**c**,**d**), and sprout number (**e**,**f**). Notes: Comparisons between species within growth traits, including sprout height, sprout basal diameter, and sprout number, were performed using the pairwise *t*-test. *** *p* < 0.001. Different letters above bars indicate statistical differences among stump diameter classes in accordance with Tukey’s honestly significant difference (HSD) mean comparison.

The effects of species and stump diameter on growth traits including sprout height, sprout diameter, and sprout number were significant (*p* < 0.001) (Table 2). All growth traits except sprout height were significantly affected by the interaction between species and stump diameter class. No significant block effect was observed in any growth traits. Pearson correlation analysis showed that the stump diameter was significantly positively correlated with the sprout height, sprout number, and sprout basal diameter in *S. suchowensis* (*p* < 0.01). A significant positive correlation was observed between the stump diameter and sprout number of *S. triandra* (*p* < 0.01). The sprout height and basal diameter regenerated from stumps of *S. triandra* were significantly higher than those of *S. suchowensis* (Figure 1a,c). However, the mean number of sprouts regenerated from *S. suchowensis* (7.8 ± 0.1) was higher than that of *S. triandra* (7.3 ± 0.09, Figure 1e). The stump in the 20–30 mm diameter class produced the highest number of sprouts (8.6 ± 0.2) among the three diameter classes (Figure 1f). The sprouts regenerated from the stumps in the 20–30 mm class showed the highest height (277.8 ± 7.3 cm, Figure 1b,d) and basal diameter (16.6 ± 0.2 mm, Figure 1d). An increase in the stump diameter led to increases in the numbers of sprouts produced per stump, the mean and dominant height and basal diameter of sprouts per stump, and the biomass of sprouts per stump.

### 3.3. Effect of Stump Diameter on Leaf Growth Traits

The effect of species on growth traits including leaf width, leaf length, and leaf area was significant (Table 2). All leaf traits except leaf area were significantly affected by stump diameter. The interaction between species and stump diameter class had no effect on the leaf traits. Moreover, no significant block effect was observed in any leaf traits. Pearson correlation analysis showed that leaf traits of *S. suchowensis and S. triandra* were correlated (Table 3). The leaf width, leaf length, and leaf area of sprouts regenerated from the stump of *S. triandra* were larger than those of *S. triandra* (Table 4). The sprouts regenerated from the stump in the 20–30 mm diameter class exhibited the largest leaf (16.87 ± 0.38 mm) and the longest leaf length (84.29 ± 4.32 mm). Pearson correlation analysis revealed that the leaf traits, including leaf width, leaf length, and leaf area, were unaffected by the stump diameter (Table 2). 

### 3.4. Effect of Stump Diameters on Leaf Chlorophyll Content

**Figure 2:**
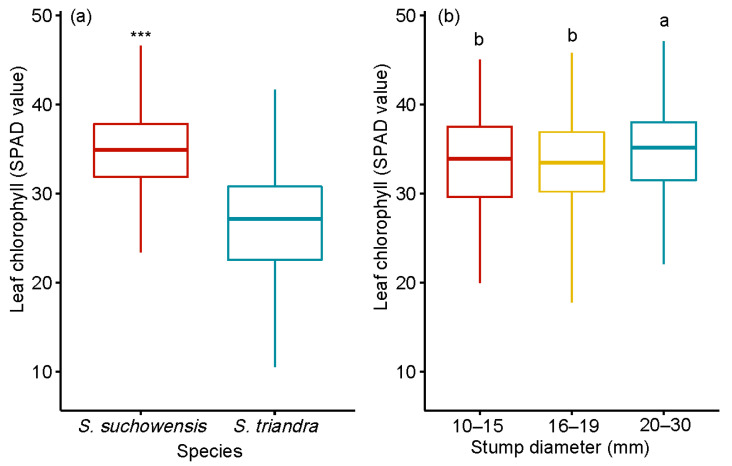
Effect of stump diameter (**a**) and species (**b**) on leaf chlorophyll content. Notes: SPAD value represented the leaf chlorophyll content. Comparisons between species and leaf chlorophyll content were performed using the pairwise *t*-test. *** *p* < 0.001. Different letters above bars indicate statistical differences among stump diameter classes in accordance with Tukey’s HSD mean comparison.

The leaf chlorophyll content of *S. suchowensis* was much higher than that of *S. triandra* (Figure 2a). The average chlorophyll content of leaves from stumps in the 20–30 mm diameter class was 34.35 ± 1.68, which was the highest (Figure 2b). Pearson correlation analysis showed that the stump diameter was significantly positively correlated with the leaf chlorophyll content in two willow species (Table 3). The effects of species and stump diameter on the leaf chlorophyll content were significant, at *p* < 0.001 (Table 2). The interaction between species and stump diameter class had no effect on leaf chlorophyll content.

### 3.5. Effect of Stump Diameters on Biomass

**Figure 3:**
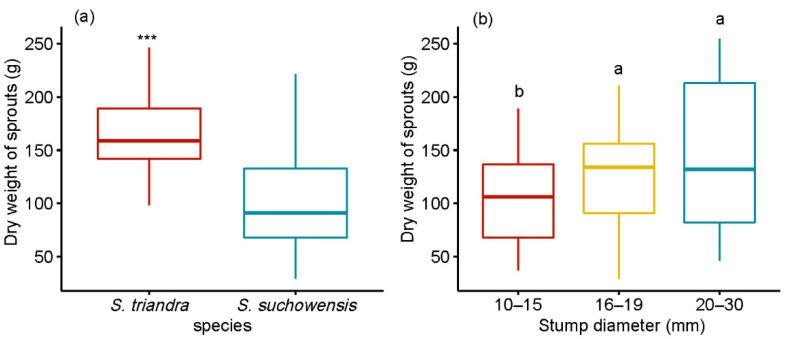
Effect of stump diameter and species on ground dry biomass of sprouts. Notes: Comparisons between species within ground dry biomass of sprouts were performed using the pairwise *t*-test. *** *p* < 0.001. Different letters above bars indicate statistical differences among stump diameter classes according to Tukey’s HSD mean comparison.

*S. triandra* performed much better than *S. suchowensis* in terms of biomass accumulation (Figure 3). The average dry weight of sprouts from stumps in the 20–30 mm diameter class was 145.9 ± 6.72 g, which was 40.77 ± 2.49 % and 18.68 ± 1.52 % heavier than those of stumps in the 10–15 and 16–19 mm classes, respectively. Pearson correlation analysis showed that the stump diameter was significantly positively correlated with the dry weight in two willow species (Table 3), and the highest correlation coefficient (r = 0.32) was observed in *S. triandra*. The effects of species and stump diameter on the dry weight of ground parts were significant, at *p* < 0.001 (Table 2). The interaction between species and stump diameter class had no effect on the dry weight.

### 3.6. Carbon and Nitrogen Proportion of the Stumps

**Figure 4:**
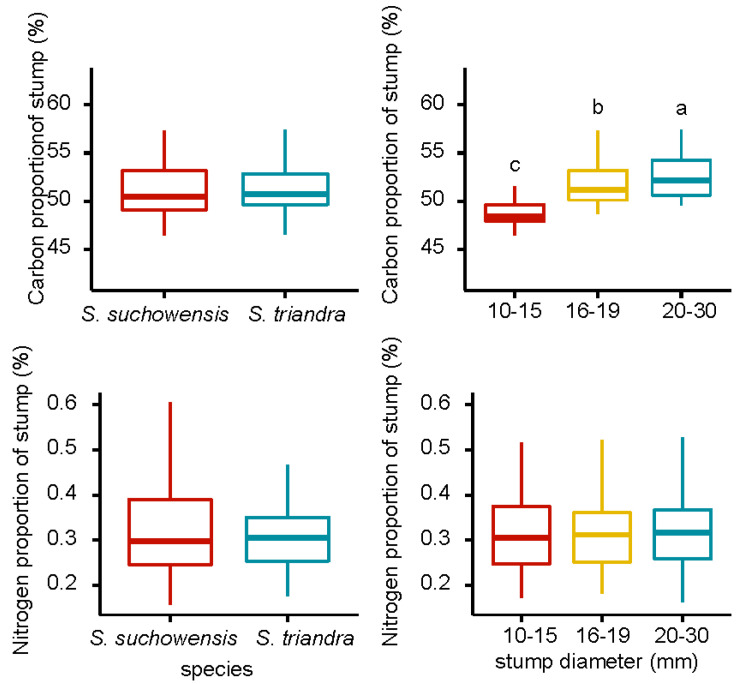
Carbon and nitrogen proportion of willow stumps. Notes: Different letters above bars indicate statistical differences among stump diameter classes according to Tukey’s HSD mean comparison.

The carbon and nitrogen proportion of stumps in *S. suchowensis* was higher than that of *S. triandra*, and the carbon and nitrogen proportion increase with an increase in stump diameter (Figure 4). Pearson correlation analysis showed that the stump diameter was significantly positively correlated with the carbon proportion in both willow species (Table 3), and the highest correlation coefficient (r = 0.63) was observed in *S. suchowensis*. The stump diameter had no effect on the nitrogen proportion in both species. The effects of species and stump diameter on the carbon proportion of stumps were significant, at *p* < 0.05 (Table 2). The interaction between species and stump diameter class had no effect on carbon and nitrogen proportion. 

### 3.7. Correlation between Growth Traits

The correlation analysis for the growth variables is listed in Table 5. Sprout height was significantly positively correlated with all the measured variables (*p* < 0.01). The strongest correlation was between leaf area and leaf length (r = 0.88). A strong correlation was also found between leaf morphological variables (leaf width, leaf length, and leaf area) and dry biomass. Leaf chlorophyll content was significantly positively correlated with sprout height, basal diameter, sprout number, and carbon proportion of stumps, but was negatively correlated with leaf width, leaf length, leaf area, and dry weight. The relation detected between sprout number and dry biomass was insignificant. 

## 4. Discussion

The survival of sprouted stumps appeared to be influenced by various factors, such as species [28], age of mother trees [17], harvest time [29], and stump size [14]. The present study showed that the stump survival in *S. suchowensis* and *S. triandra* was over 96%. The survival rate of the stumps of these two shrub willows after the first-rotation harvest was much higher than that reported for oak coppice, at 84–86% [30], or short-rotation eucalypt coppice, at 81–91% [31]. This finding proves that these two shrub willows have great potential to become commercial short-rotation tree species. Several studies have found a negative effect of stump diameter on stump survival [32,33]. A converse tendency in stump survival was observed in other species [17,18,34]. However, the stump diameter had no significant effect on stump survival in the present study. Furthermore, the causes of high mortality of stumps after cutting are often related to the aging of stumps and inappropriate cutting seasons [30,31]. Old tree stumps with large diameters usually have large-sized and aged root systems, which may lead to an imbalance of resources between the root systems and sprouts. In addition, the resources produced by sprouts are insufficient to maintain the root system, which may be the possible cause of death of these stumps. Cutting in the dormant season could minimize the mortality rate and produce more buds [35], whereas cutting before budding could increase stump mortality [28]. In the present study, the stumps used in the field trial were cut during the dormant period, and the stumps were only 2 years old, which accounts for the low mortality observed in these two willow stumps. Therefore, the low mortality levels recorded in this study may be considered typical of a healthy coppice forest managed in accordance with good practices.

The growth status of sprouts is an important indicator that determines the competitive success of stump sprouts with neighboring plants [13,36]. The present study suggests that the willow stump diameter has a great effect on the re-sprouting of coppiced stumps. An increase in stump diameter led to increased numbers of sprouts produced per stump, as well as in the mean and dominant height and basal diameter of sprouts per stump in *S. suchowensis* and *S. triandra*. This result was similar to that of Ashish et al. [37] and Randall et al. [38]. However, the effect of stump diameter on sprouting has been quite variable in other studies. Dinh et al. [34] observed that the growth parameters (diameter and length) of dominant sprouts were not significantly affected by stump diameter in two oak trees. Hytönen [17] found that the number of sprouts per living stump did not increase any more when the stump diameter in the two oldest age classes exceeded 5 cm. Stumps and root systems are the main sources of carbon for tree sprouting and regeneration [39,40]. The positive relationship between sprouting ability and stump diameter may be attributed to larger stumps having a larger surface and much more complete root system and storing more available carbohydrates than small-sized stumps [41,42]. Similar to these prior findings [16,41], our data indicate that the larger stumps store more carbon and nitrogen than small-sized stumps, which may be one of the reasons why the larger willow stumps have a stronger resprouting ability. Larger stumps could provide more space and energy for sprouts and produce more sprouts when self-thinning occurs during the second growing seasons after cutting. In addition, the age of the stump is a key factor that affects its regeneration. Despite having similar stump diameters, younger trees produced taller sprouts than older trees [14,17].

The biomass of sprouts on a stump increased with the increase in stump diameter in oak species [43]. In the present study, stump diameters were significantly positively correlated with the biomass of sprouts per stump. By contrast, Hytönen et al. [17] found no significant correlation between the stump diameter and the biomass of sprouts when the trees were older than a certain age. This opposite tendency could be attributed to the weakened sprouting ability in older trees. In addition, in the same diameter group, the dry biomass of sprouts per stump of *S. triandra* was larger than that of *S. suchowensis*. This result provides evidence that *S. triandra* has a stronger regeneration ability after cutting than *S. suchowensis*. A positive correlation was observed between leaf morphological variables (leaf width, leaf length, and leaf area) and the dry biomass of sprouts per stump. Leaf area is closely related to biomass [44], which is important for plant growth. The growth of the leaf area determines the interception of light, and this is an important parameter that determines plant productivity [45,46]. In the present study, the leaf area of *S. triandra* was significantly larger than that of *S. suchowensis*. This indicates that the photosynthetic capacity of *S. triandra* stump sprouts may be higher than that of *S. suchowensis.* No significant correlation between leaf area and stump diameter was observed in this study. However, the stump diameter had a significantly positive effect on the leaf chlorophyll content. Chlorophyll is a major determinant that reflects the plant’s photosynthetic ability [47]. The leaves on sprouts produced by large stumps have higher photosynthetic efficiency than those produced by small stumps, which may also be one of the reasons for the higher biomass produced by larger stumps.

## 5. Conclusions

The diameter of stumps has a strong positive influence on the regeneration ability of two shrub willows. The results also reveal that larger stumps store more carbon and nitrogen than small stumps, which may be one of the reasons why larger willow stumps have a stronger resprouting ability. This study provides fundamental information for the management of SRC plantations and the optimal cultivation patterns of willows.

## Figures and Tables

**Table 1:** Number and the survival rate of different stump species and diameter classes.

Species	Stump Diameter Classes	Stump Number	Survival Rate (%)
*S. suchowensis*	10–15	1215	96.13
	16–19	1476	96.50
	20–30	1209	98.84
	total	3900	96.51
*S. triandra*	10–15	165	94.29
	16–19	267	94.68
	20–30	348	98.58
	total	780	96.15

**Table 2:** Mixed model ANOVA results for growth variables, including source of variation, degrees of freedom (df), and F-values.

		Fixed Effects		Random Effects	
Trait	Species	Diameter Class	Interaction	Block	Error
	(df = 1)	(df = 2)	(df = 2)	σ^2^ (%)	σ^2^ (%)
Sprout height	21.84 ***	20.68 ***	3.48 *	3.10	96.90
Basal diameter	25.82 ***	24.19 ***	1.41	16.10	83.90
Sprout number	18.52 ***	40.25 ***	1.21	1.40	98.60
Leaf width	359.30 ***	5.85 **	1.52	3.47	96.53
Leaf length	635.09 ***	3.24 *	0.69	0	100.00
Leaf area	347.53 ***	0.38	0.40	0.10	99.90
Leaf chlorophyll	618.16 ***	11.98 ***	0.45	8.89	91.11
Dry weight	100.92 ***	18.13 ***	0.19	0	100.00
Carbon content of stump	3.30 *	30.93 ***	0.46	3.50	96.50
Nitrogen content of stump	0.14	0.81	0.84	10.20	89.80

Notes: The value of the fixed factor represents the F value * *p* < 0.05, ** *p* < 0.01, *** *p* < 0.001. σ^2^, variance component.

**Table 3:** Pearson correlation coefficients (r) between stump diameter and growth traits in two willow species.

Growth Traits	*S. suchowensis*	*S. triandra*
	Stump Diameter	
Sprout height	0.46 **	0.11
Basal diameter	0.40 **	0.22
Sprout number	0.36 **	0.35 **
Leaf width	0.27	0.20
Leaf length	0.15	0.01
Leaf area	0.27	0.13
Leaf chlorophyll	0.19 **	0.16 **
Dry weight	0.31 *	0.32 **
Carbon content of stump	0.63 **	0.16 *
Nitrogen content of stump	0.03	0.06

Notes: The values represent correlation coefficients. * *p* < 0.05, ** *p* < 0.01.

**Table 4:** Effect of stump diameter on leaf traits.

Species	Stump Diameter (mm)	Leaf Width (mm)	Leaf Length (mm)	Leaf Area (mm^2^)
*S. suchowensis*	10–15	11.20 ± 0.28	54.11 ± 2.52	633.31 ±15.58
	16–19	11.56 ± 0.21	50.36 ± 2.64	610.05 ± 16.75
	20–30	11.78 ± 0.40	53.94 ± 2.60	607.30 ± 12.22
	Mean	11.52 ± 0.24	52.64 ± 2.97	616.11 ± 14.59
*S. triandra*	10–15	16.59 ± 0.26	81.35 ± 4.08	962.94 ± 13.12
	16–19	16.40 ± 0.39	81.01 ± 4.53	964.92 ± 19.77
	20–30	16.87 ± 0.38	84.29 ± 4.32	968.05 ± 16.89
	Mean	16.65 ± 0.35	82.55 ± 4.17	965.31 ± 14.95

Notes: The results are represented as mean ± SE. SE, standard error.

**Table 5:** Pearson correlation coefficients (r) among growth traits.

Growth Traits	Height	Basal Diameter	Sprout Number	Leaf Width	Leaf Length	Leaf Area	Dry Weight	Leaf Chlorophyll
Height	1.00							
Basal diameter	0.81 **	1.00						
Branch number	0.51 **	0.50 **	1.00					
Leaf width	0.26 **	0.21 **	0.03	1.00				
Leaf length	0.21 **	0.22 **	0.07	0.64 **	1.00			
Leaf area	0.25 **	0.21 **	0.03	0.82 **	0.88 **	1.00		
Dry weight	0.42 **	0.33 **	0.13	0.32 **	0.26 **	0.35 **	1.00	
Leaf chlorophyll	0.16 **	0.16 **	0.41 **	−0.29 **	−0.23 **	−0.20 **	−0.24 **	1.00

Notes: ** *p* < 0.01.
